# Risk factors and a prediction model for unruptured intracranial aneurysms in patients with ischemic stroke using carotid intima-media thickness and systemic atherosclerosis

**DOI:** 10.3389/fneur.2023.1227673

**Published:** 2023-08-29

**Authors:** Gaiming Gao, Dezhi Kang, Jinning Zhang, Zhixian Jiang, Xueyang He, Yanyu Wu

**Affiliations:** ^1^Department of Neurosurgery, First Hospital of Quanzhou, Quanzhou, Fujian, China; ^2^Department of Neurosurgery, The First Affiliated Hospital of Fujian Medical University, Fuzhou, Fujian, China; ^3^Department of Anesthesiology, First Hospital of Quanzhou, Quanzhou, Fujian, China

**Keywords:** carotid intima-media thickness, unruptured intracranial aneurysms, systemic atherosclerosis, ischemic stroke, risk factors, prediction model

## Abstract

**Background:**

Systemic atherosclerosis and carotid intima-media thickness (IMT) have been widely used in clinical practice for ischemic stroke; however, little is known about the risk factors for unruptured intracranial aneurysms (UIAs) in patients with ischemic stroke (IS). Therefore, we performed this study to identify the risk factors and construct a prediction model for UIA in patients with IS.

**Methods:**

Data were retrospectively collected from patients with IS from 2015 to 2022 at the First Hospital of Quanzhou City, Quanzhou, Fujian, China. Risk factors for UIA in patients with IS were identified using a multivariate logistic regression model, and a receiver operating characteristic (ROC) curve was applied to construct the prediction model.

**Results:**

Out of the 122 patients with IS, 52 who presented with UIA (ISUIA) were categorized into the study group and the remaining 70 IS patients without UIA into the control group. Patients in the ISUIA group had lower carotid IMT and carotid artery plaque scores than those in the IS group (*P* < 0.05). Multivariate analyses found that aspirin use (OR: 12.987; *P* = 0.031), elevated C-reactive protein (CRP) level (OR: 1.019; *P* = 0.004), and carotid IMT > 0.09 mm (OR: 0.218; *P* < 0.001) were significantly associated with the risk of UIA in patients with IS. However, UIA in patients with IS was unaffected by the carotid artery plaque score (*P* = 0.114). The constricted prediction model based on the abovementioned factors for UIA in IS patients was 0.79 (95% CI: 0.71–0.87).

**Conclusion:**

The findings revealed that the risk factors for UIA in patients with IS included aspirin use, elevated CRP level, and smaller carotid IMT, and the predictive value of the prediction model was relatively better.

## Introduction

Unruptured intracranial aneurysm (UIA) is a chronic cerebrovascular disorder with an estimated prevalence of 2.3–3.2% (1, 2), is considered the main cause of subarachnoid hemorrhage (SAH), and is associated with high fatality and morbidity (3). In clinical practice, UIA is difficult to detect owing to its asymptomatic nature and is incidentally discovered on medical images (4). UIA is diagnosed during the onset of ischemic stroke (IS) or transient ischemic attack (5). The clinical management of incidentally identified UIA remains controversial because most UIAs are small, with diameters of <5 mm (5–7). However, the prevalence of ruptured intracranial aneurysms showing SAH in UIA <5 mm is up to 47% (8). Therefore, UIA should be identified promptly for further risk assessment in patients with IS.

Carotid intima-media thickness (IMT), measured using B-mode ultrasound, is considered a sensitive, non-invasive, reproducible, and well-validated method for detecting early atherosclerotic changes within the arterial walls. It is significantly associated with systemic atherosclerosis and future cardiovascular events (9). The thickening of the arterial wall can be observed before the narrowing of the lumen and symptoms of the disease (10). Studies have previously demonstrated that carotid IMT is significantly associated with carotid plaque development, cardiovascular risk, and cognitive function (11–13). Moreover, a previous study found that the incidence of intracranial aneurysms in patients with IS was 6.6%, and the risk factors included female sex and old age (14). However, whether UIA in patients with IS could be affected by carotid IMT and systemic atherosclerosis and the prediction model for UIA in patients with IS were not addressed. Therefore, we performed this study to identify the potential risk factors and construct a predictive model for UIA in patients with IS based on carotid IMT, systemic atherosclerosis, and clinical factors.

## Methods

### Study design and population

A total of 52 patients with IS diagnosed with UIA between 2015 and 2022 at the First Hospital of Quanzhou City, Quanzhou, Fujian, China were enrolled in this study. Additionally, 70 patients diagnosed with non-cardiac embolism IS without any presenting symptoms of UIA were recruited as the control group. The inclusion criteria were as follows: (1) adult patients aged > 18.0 years; (2) IS diagnosed according to the China Acute Ischemic Stroke Diagnosis and Treatment Guidelines 2014 (15); and (3) available data on carotid IMT, systemic atherosclerosis, and clinical factors. Patients were excluded if they (1) had a history of SAH, brain trauma, intracranial hemorrhage, brain infection, or space-occupying lesions; (2) had other serious systemic diseases; or (3) had incomplete information regarding UIA, carotid IMT, and systemic atherosclerosis. The Institutional Review Board of the First Affiliated Hospital of Fujian Medical University approved this study. Informed consent was not required because of the retrospective nature of the study.

### Carotid ultrasound

B-mode carotid ultrasound was performed using a Sonosite MicroMaxx Portable Ultrasound with a 13–6 MHz linear array transducer. Carotid ultrasonograms of all included patients were assessed by the same carotid sonographer after professional training. Patients were placed comfortably in the supine position, with the neck slightly extended and the head directed slightly away from the side of interest, then the longitudinal images of the right and left proximal, mid, and distal common carotid artery were obtained. Proximal was defined as the image of the artery obtained when the transducer was placed as close to the heart as possible (usually at the base of the neck, just above the clavicle). Distal was defined as the image of the artery obtained with the carotid bulb visible to the far left of the screen. The middle image is obtained between the proximal and distal sections. All images were recorded during diastole. The transducer was placed at several angles (anterior, lateral, and posterior), and the CCA image that provided the best view of the intima/media layer was selected. The anterior angle (the neck area parallel to the trachea, several centimeters off the midline, and perpendicular to the mattress) often provides the best image.

### Carotid IMT

A technician measured the IMT using Sonosite SonoCalc IMT software, a computer-based algorithm with a manual override. Measurements were performed on the far wall of the CCA with a segment length of 10 mm. The thickest section in the image was selected for the measurement. Plaque, defined as an area that demonstrates a thickness of ≧1.5 mm, was incorporated in the average. The “auto” function was first used as Sonosite SonoCalc software, which was recommended. If the fit was inaccurate, the “sketch” function was selected, which allowed for better approximation. The functions approximate the lumen–intima and media–adventitia interfaces and apply an automatic manual override technique to calculate the IMT, which is the distance between the two interfaces. IMT measurements were performed from stored images randomly accessed months or years after acquisition, so the technician did not know whether the image belonged to a patient or a control.

### Carotid artery plaque scores

In participants with plaque, the total number of sites with plaques was calculated and ranged from one to four (left and right CCAs and CB-ICAs). A semi-quantitative scale score was also used to assess the severity of the plaque. It was graded as follows: 1 site (left or right) with plaques having thickness of ≤ 2 mm (score = 1), 2 sites with plaques with both thicknesses of ≤ 2 mm, or 1 site with plaques having thickness of >2 mm (score = 2), 2 sites with plaques including at least 1 plaque with thickness of >2 mm (score = 3), and 2 sites with plaques with both thicknesses of >2 mm or annular plaque (score = 4) (16).

### Clinical variables

The clinical characteristics of identified patients were obtained from electronic medical records, including sex, age, hypertension (antihypertensive drug treatment or physician diagnosis), diabetes mellitus (DM, medications, or physician diagnosis), family history (at least one family member with cardiovascular disease, hypertension, hyperlipidemia, or DM), smoking status, alcohol intake, history of spider blood disease, aspirin use, hyperlipidemia, hyperfibrinogenemia, hyperhomocysteinemia, lymphocyte percentage, C-reactive protein (CRP), history of IS, and history of coronary heart disease (CHD).

### UIA measurement

Magnetic resonance imaging (MRI) of the head (Siemens Magnetom Verio 3.0 T MRI, Siemens, Erlangen, Germany) and digital subtraction angiography (DSA) (Siemens, Erlangen, Germany) were used to assess the shape, location, and size of the UIA. The shape of the UIA is classified into capsular and fusiform aneurysms. In contrast, the anatomical location of UIA is classified into the cavernous part of the carotid artery (CpCA), the internal carotid artery (ICA), the anterior communicating or anterior cerebral artery (ACA), the middle cerebral artery (MCA), the posterior communicating artery (PCA), and the vertebrobasilar system (VB). The size of UIA was classified into 2–7, 8–12, 13–24, and 25 mm.

### Statistical analysis

Carotid IMT, systemic atherosclerosis, and clinical factors were assigned as continuous and categorical data. Mean (standard deviation) or median (interquartile range) was used to describe continuous data based on data distribution and whether it met normal distribution. In contrast, numbers and percentages were used to describe categorical data. Differences between the ISUIA and IS groups were compared using the independent *t*-test, the Kruskal–Wallis test, or the chi-squared test. The risk factors for UIA in patients with IS were identified using univariate logistic regression analysis. The potential risk factors were subjected to the multivariate logistic regression analysis using α = 0.05 and β = 0.10. The effect estimates for each factor were presented as odds ratios (OR) and 95% confidence intervals (CIs). A receiver operating characteristic (ROC) curve was used to construct a predictive model for UIA in patients with IS. The predictive value was assessed using the area under the curve (AUC). All reported *P*-values were two-sided, and the inspection level was 0.05. All statistical analyses were performed using SPSS version 26 for Windows (SPSS, Armonk, NY, USA).

## Results

### Baseline characteristics

The baseline characteristics of the ISUIA and IS groups are shown in Table 1, comprising 122 patients, with 52 in the ISUIA group and 70 in the IS group. The median age of the patients was 60.50 years, with 68.03% being male. There were no significant differences between the two groups in terms of sex (*P* = 0.731), age (*P* = 0.467), hypertension (*P* = 0.433), DM (*P* = 1.000), family history (*P* = 0.575), smoking status (*P* = 0.524), alcohol intake (*P* = 0.322), history of spider blood disease (*P* = 0.635), hyperlipidemia (*P* = 0.363), hyperfibrinogenemia (*P* = 0.094), hyperhomocysteinemia (*P* = 1.000), lymphocyte percentage (*P* = 0.188), history of IS (*P* = 0.705), and history of CHD (*P* = 0.514). However, we noted significant differences between the ISUIA and IS groups for aspirin use (*P* = 0.041) and CRP (*P* < 0.001).

**Table 1:** Baseline characteristics of included patients.

**Variables**	**Overall (*n* = 122)**	**Group**		***P*-value**
		**IS (*****n*** = **70)**	**ISUIA (*****n*** = **52)**	
Sex (%)				0.731
Male	83 (68.03)	49 (70.00)	34 (65.38)	
Female	39 (31.97)	21 (30.00)	18 (34.62)	
Age	60.50 (53.25, 70.00)	60.00 (55.00, 69.00)	61.00 (49.50, 70.00)	0.467
Hypertension (%)				0.433
Yes	81 (66.39)	49 (70.00)	32 (61.54)	
No	41 (33.61)	21 (30.00)	20 (38.46)	
Diabetes mellitus (%)				1.000
Yes	26 (21.31)	15 (21.43)	11 (21.15)	
No	96 (78.69)	55 (78.57)	41 (78.85)	
Family history (%)				0.575
Yes	3 (2.46)	1 (1.43)	2 (3.85)	
No	119 (97.54)	69 (98.57)	50 (96.15)	
Smoking status (%)				0.524
Yes	45 (36.89)	28 (40.00)	17 (32.69)	
No	77 (63.11)	42 (60.00)	35 (67.31)	
Alcohol intake (%)				0.322
Yes	10 (8.20)	4 (5.71)	6 (11.54)	
No	112 (91.80)	66 (94.29)	46 (88.46)	
History of spider blood disease (%)				0.635
Yes	4 (3.28)	3 (4.29)	1 (1.92)	
No	118 (96.72)	67 (95.71)	51 (98.08)	
Aspirin use (%)				0.041
Yes	7 (5.74)	1 (1.43)	6 (11.54)	
No	115 (94.26)	69 (98.57)	46 (88.46)	
Hyperlipidemia (%)				0.363
Yes	38 (31.15)	19 (27.14)	19 (36.54)	
No	84 (68.85)	51 (72.86)	33 (63.46)	
Hyperfibrinogenemia (%)				0.094
Yes	21 (17.21)	16 (22.86)	5 (9.62)	
No	101 (82.79)	54 (77.14)	47 (90.38)	
Hyperhomocysteinemia (%)				1.000
Yes	26 (21.31)	15 (21.43)	11 (21.15)	
No	96 (78.69)	55 (78.57)	41 (78.85)	
Lymphocyte percentage (%)	19.20 (15.12, 25.80)	19.85 (16.30, 28.42)	18.95 (14.38, 23.55)	0.188
CRP	11.33 (2.06, 27.65)	3.48 (1.17, 11.79)	23.15 (12.23, 36.60)	<0.001
History of IS (%)				0.705
Yes	12 (9.84)	8 (11.43)	4 (7.69)	
No	110 (90.16)	62 (88.57)	48 (92.31)	
History of CHD (%)				0.514
Yes	10 (8.20)	7 (10.00)	3 (5.77)	
No	112 (91.80)	63 (90.00)	49 (94.23)	
Carotid artery plaque score	1.00 (0.00, 2.00)	1.00 (0.00, 3.00)	1.00 (0.00, 2.00)	0.017
IMT (%)				<0.001
≤ 0.09 mm	52 (42.62)	19 (27.14)	33 (63.46)	
>0.09 mm	70 (57.38)	51 (72.86)	19 (36.54)	

### Clinical characteristics of UIA

The shape, anatomical location, and size of the UIA are summarized in Table 2. Overall, we noted that 88.5% (46/52) of the patients had capsular aneurysms, and UIA was mostly located in the CpCA (38.5%), ACA (21.2%), and ICA (15.4%). Moreover, 71.2% of the patients had a UIA ranging from 2 to 7 mm, and 17.3% had a UIA ranging from 8 to 12 mm.

**Table 2:** Clinical characteristics of UIA in IS patients.

**UIA**	***N* (%)**
**Shape of aneurysms**	
Capsular aneurysm	46 (88.5%)
Fusiform aneurysm	6 (11.5%)
**Anatomical location of UIA**	
Cavernous part of the carotid artery	20 (38.5%)
Internal carotid artery	8 (15.4%)
Anterior communicating or anterior cerebral artery	11 (21.2%)
Middle cerebral artery	4 (7.7%)
Posterior communicating artery	4 (7.7%)
Vertebrobasilar system	5 (9.6%)
**Size of UIA (in mm)**	
2–7	37 (71.2%)
8–12	9 (17.3%)
13–24	4 (7.7%)
≧25	2 (3.8%)

### Carotid IMT and carotid artery plaque scores

**Figure 1:**
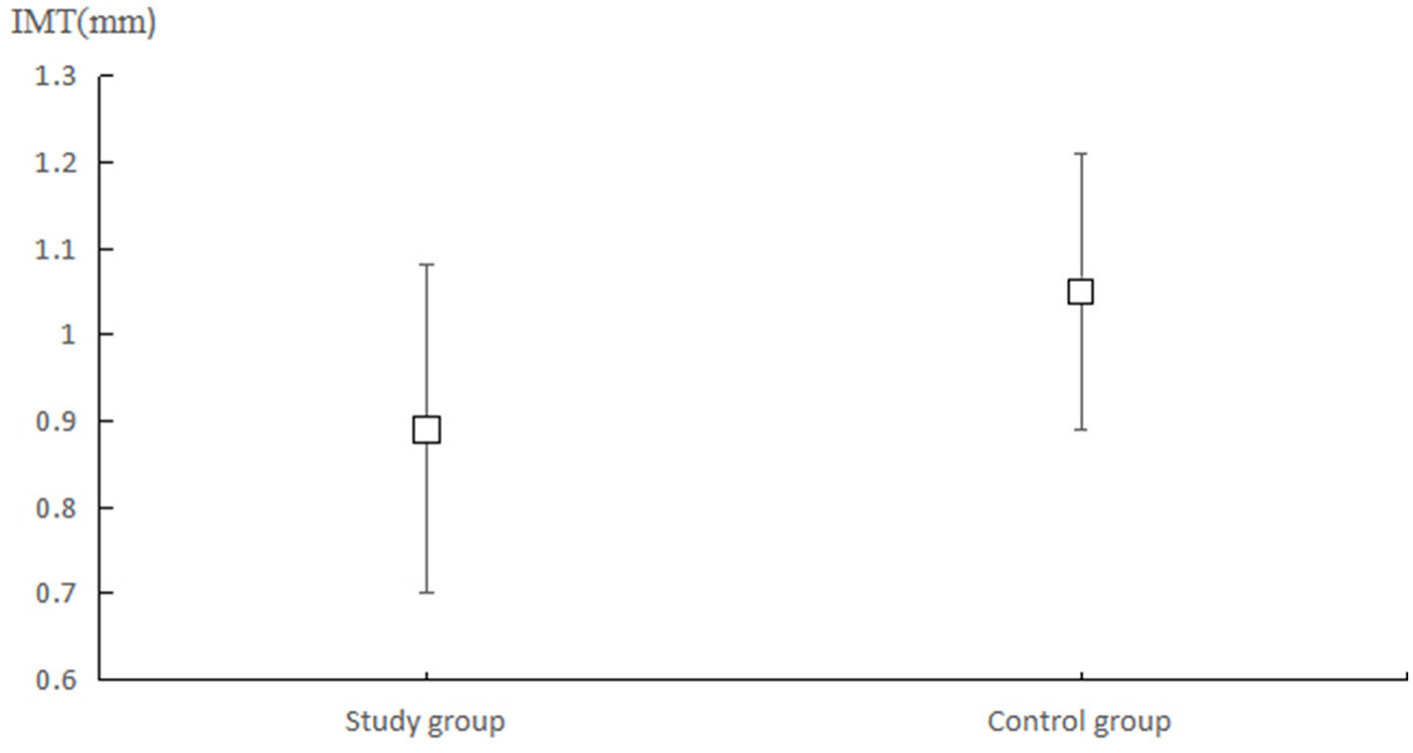
Mean carotid IMT in the ISUIA and IS groups.

The mean carotid IMT in the ISUIA and IS groups were 0.89 (0.19) and 1.00 (0.16) mm (Figure 1), while the carotid artery plaque scores in the ISUIA and IS groups were 1.04 (1.44) and 1.76 (1.853), respectively. The patients in the ISUIA group had lower carotid IMT (*P* = 0.001) and carotid artery plaque scores (*P* = 0.022). Considering that the carotid IMT and carotid artery plaque scores were not normally distributed, we noted significant differences between the ISUIA and IS groups for carotid IMT (*P* < 0.001) and carotid artery plaque scores (*P* = 0.017).

### Risk factors for UIA in IS patients

The univariate and multivariate logistic regression analyses of UIA in patients with IS are shown in Table 3. Univariate analysis found that aspirin use (OR: 9.009; 95% CI: 1.048–76.923; *P* = 0.045) and elevated CRP (OR: 1.015; 95% CI: 1.002–1.027; *P* = 0.024) were associated with an increased risk of UIA in patients with IS, while elevated carotid artery plaque scores (OR: 0.751; 95% CI: 0.582–0.969; *P* = 0.028) and carotid IMT > 0.09 mm (OR: 0.214; 95% CI: 0.099–0.464; *P* < 0.001) were associated with a reduced risk of UIA in IS patients. We noted that aspirin use (OR: 12.987; 95% CI: 1.261–125.000; *P* = 0.031) and elevated CRP (OR: 1.019; 95% CI: 1.006–1.032; *P* = 0.004) were associated with an increased risk of UIA, while carotid IMT > 0.09 mm was associated with a reduced risk of UIA in patients with IS (OR: 0.218; 95% CI: 0.092–0.516; *P* < 0.001), after adjusting for potential confounding factors. However, the carotid artery plaque score was not associated with the risk of UIA in patients with IS (OR: 0.805; 95% CI: 0.616–1.053; *P* = 0.114).

**Table 3:** Univariate and multivariate logistic regression analyses for UIA in IS patients.

**Variable**	**Univariate**		**Multivariate**	
	**OR**	***P-*** **value**	**OR**	***P-*** **value**
**Sex (%)**				
Male	1			
Female	1.235 (0.574–2.659)	0.589		
Age	0.981 (0.952–1.011)	0.207		
**Hypertension (%)**				
No	1			
Yes	0.686 (0.221–1.462)	0.329		
**Diabetes mellitus (%)**				
No	1			
Yes	0.983 (0.409–2.364)	0.971		
**Family history of UIA (%)**				
No	1			
Yes	2.762 (0.243–31.250)	0.413		
**Smoking status (%)**				
No	1			
Yes	0.728 (0.344–1.543)	0.409		
**Alcohol intake (%)**				
No	1			
Yes	2.151 (0.575–8.065)	0.255		
**History of spider blood disease (%)**				
No	1			
Yes	0.438 (0.044–4.329)	0.480		
**Aspirin use (%)**				
No	1			
Yes	9.009 (1.048–76.923)	0.045	12.987 (1.261–125.000)	0.031
**Hyperlipidemia (%)**				
No	1			
Yes	1.546 (0.714–3.344)	0.269		
**Hyperfibrinogenemia (%)**				
No	1			
Yes	0.359 (0.122–1.055)	0.063		
**Hyperhomocysteinemia (%)**				
No	1			
Yes	0.983 (0.409–2.364)	0.971		
Lymphocyte percentage (%)	0.969 (0.931–1.009)	0.127		
CRP	1.015 (1.002–1.027)	0.024	1.019 (1.006–1.032)	0.004
**History of IS (%)**				
No	1			
Yes	0.646 (0.184–2.273)	0.496		
**History of CHD (%)**				
No	1			
Yes	0.551 (0.136–2.242)	0.405		
Carotid artery plaque scores	0.751 (0.582–0.969)	0.028	0.805 (0.616–1.053)	0.114
**IMT (%)**				
≤ 0.09 mm	1			
>0.09 mm	0.214 (0.099–0.464)	<0.001	0.218 (0.092–0.516)	<0.001

### Prediction model

**Figure 2:**
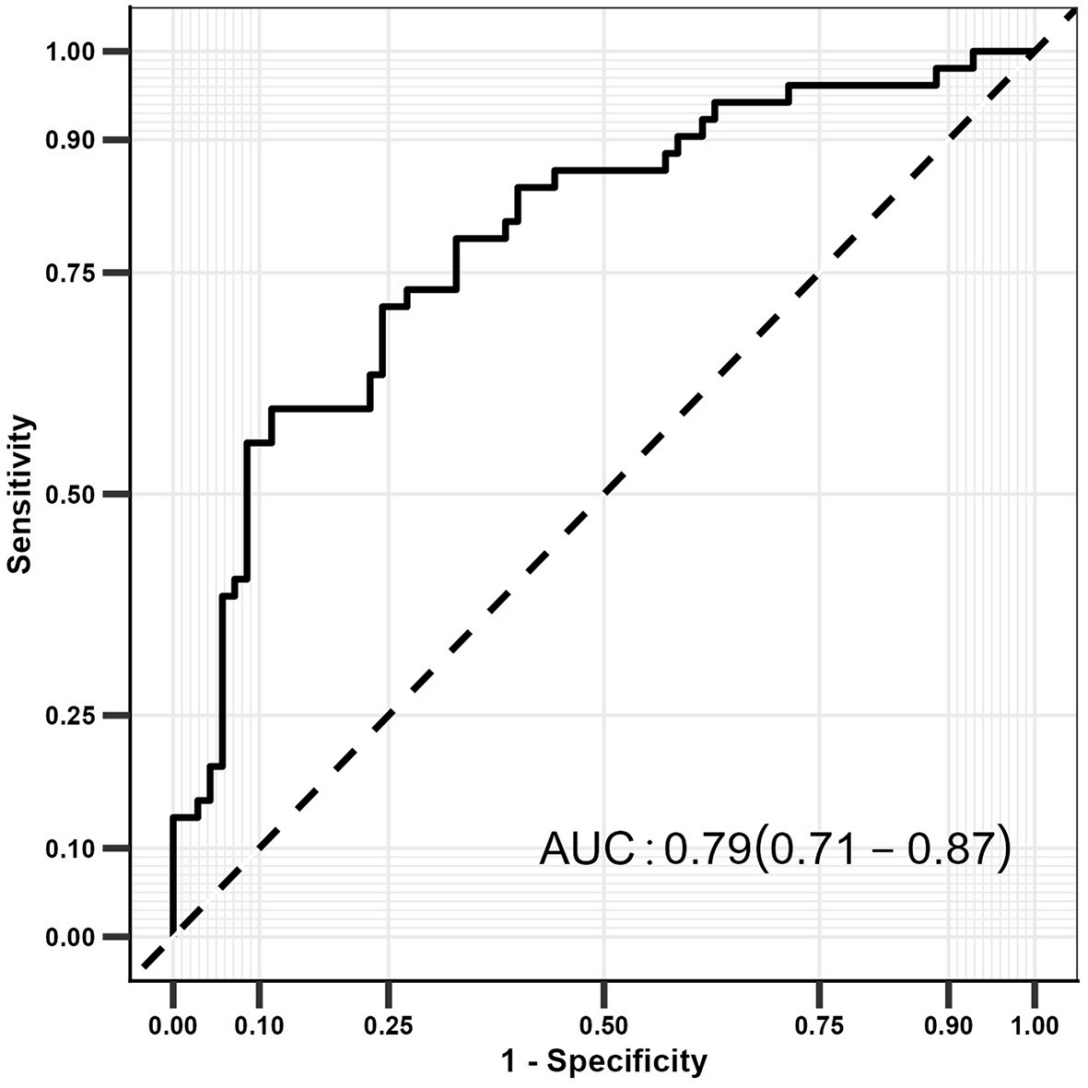
The ROC curve for UIA in patients with IS.

The prediction model was constructed according to the results of the multivariate logistic regression analysis, and the model constructed included aspirin use, CRP, carotid artery plaque score, and carotid IMT score (Figure 2). The constructed model had a relatively high predictive value for detecting UIA in patients with IS (AUC: 0.79; 95% CI: 0.71–0.87).

## Discussion

This study systematically identified the risk factors for UIA in patients with IS. A multifactorial predictive model was constructed to identify patients with IS at high risk for UIA. This retrospective study included 122 patients with IS and 52 patients with UIA. Patients in the ISUIA group had lower carotid IMT and carotid artery plaque scores than those in the IS group. Multivariate analysis indicated that the risk of UIA in patients with IS could be affected by aspirin use, elevated CRP, and carotid IMT > 0.09 mm. In contrast, the carotid artery plaque score was not associated with the risk of UIA in patients with IS. The constructed prediction model based on aspirin use, CRP level, carotid IMT, and carotid artery plaque scores showed relatively better predictive performance for UIA in patients with IS.

Several studies have reported incidental intracranial aneurysms in patients (17–19). Oh et al. collected data from 314 patients with IS, and the prevalence of incidental intracranial aneurysms was 6.1%; the presence of incidental intracranial aneurysms in IS patients was more evident in female and older patients (17). Jiranukool et al. reported that 7% of 186 patients with IS presented with incidental intracranial aneurysms, and no significant risk factors were identified. Moreover, they found that IS patients presented with a higher prevalence of intracranial aneurysms than the general population, while IS patients who had intracranial aneurysms did not have an effect on functional outcomes (18). Wu et al. retrospectively collected data from 4,033 patients with IS and found that 6.5% of patients presented with UIA, and incidental UIA was not associated with the prognosis of IS (19). However, the risk factors and predictive models for UIA in patients with IS remain unclear. Therefore, we performed this study to identify the risk factors and construct a prediction model for UIA in patients with IS.

This study found that patients with IS and UIA had lower carotid IMT and carotid artery plaque scores than those with IS alone. A previous study found that carotid IMT was smaller in patients with ascending aortic aneurysms and that patients with ascending aortic aneurysms could protect against the progression of atherosclerosis (20). The excess proteolytic balance of matrix metalloproteinase activity and the transformation of growth factor β contributed an important role for the association between the pro-aneurysmal state conferring protection and atherosclerosis (21–24). The mechanism of formation is different in aneurysms at various positions owing to the embryological origin of different parts of the aorta (25, 26).

Our study found that UIA in patients with IS could be affected by aspirin use, CRP level, and carotid IMT, while the carotid artery plaque score did not affect the risk of UIA. Several reasons could explain these results: (1) Aspirin is widely used for patients at high risk of cardiovascular disease and stroke, which is significantly related to the progression of UIA (17, 27); (2) CRP can immediately respond to the inflammatory state, and serum CRP will increase rapidly due to the aggravation of the disease (28, 29). Moreover, the inflammatory state could promote endothelial cell dysfunction, which is significantly associated with the progression of abdominal aortic aneurysms (30, 31); and (3) carotid IMT reflects the thickness of the intima-medial layers of the vessel wall, which is significantly related to the arterial injury and inflammatory process at an early stage (32). In addition, the prediction model based on aspirin use, CRP, carotid IMT, and carotid artery plaque scores had a relatively better predictive performance for UIA in patients with IS, which suggests that the constructed model could screen IS patients who are at high risk for UIA. Therefore, effective treatments should be used to improve the prognosis of UIA in patients with IS.

This study had several limitations. First, this was a retrospective study, and the causality of risk factors for UIA in patients with IS could not be determined. Second, the background therapies for IS differed among the included patients, which might have played an important role in the progression of UIA. Third, the severity of the UIA was not addressed in the constructed prediction model, and the prognosis differed for UIA of various shapes, anatomical locations, and sizes. Fourth, the constructed prediction model was not validated in an external cohort, and the results of this study should be cautiously recommended.

## Conclusion

This study found that patients with IS and UIA had lower carotid IMT and carotid artery plaque scores. We noted that aspirin use, elevated CRP, and carotid IMT > 0.09 mm were significantly associated with the risk of UIA after adjusting for potential confounding factors. In contrast, the carotid artery plaque score was not associated with the risk of UIA in patients with IS. The prediction model constructed based on these factors exhibited relatively better predictive performance. Further large-scale prospective studies should be conducted to verify the results of this study and validate the reliability of the constructed prediction model.

## Data availability statement

The raw data supporting the conclusions of this article will be made available by the authors, without undue reservation.

## Ethics statement

This study was approved by the Institutional Review Board of the First Affiliated Hospital of Fujian Medical University. The study was conducted in accordance with the local legislation and institutional requirements.

## Author contributions

GG and YW mainly participated in the literature search, study design, writing, and critical revision and prepared Figures 1, 2. DK, JZ, and ZJ mainly participated in data collection, data analysis, and data interpretation and prepared Tables 1–3. XH is responsible for supervision work. All authors have read and approved the final manuscript.
